# Editorial: Human movement in simulated hypogravity – Bridging the gap between space research and terrestrial rehabilitation

**DOI:** 10.3389/fneur.2024.1520540

**Published:** 2024-12-19

**Authors:** David Andrew Green, Nolan Herssens, Daniel Ciampi de Andrade, Tobias Weber, Enrico De Martino

**Affiliations:** ^1^KBR, Wyle Laboratories GmbH, Cologne, Germany; ^2^Centre of Human and Applied Physiological Sciences, King's College London, London, United Kingdom; ^3^Institute for Risk and Disaster Reduction, University College London, London, United Kingdom; ^4^Space Medicine Team, European Astronaut Centre, European Space Agency, Cologne, Germany; ^5^Department of Health Science and Technology, Center for Neuroplasticity and Pain, Faculty of Medicine, Aalborg University, Aalborg, Denmark; ^6^Aerospace Medicine and Rehabilitation Laboratory, Faculty of Health and Life Sciences, Northumbria University, Newcastle upon Tyne, United Kingdom

**Keywords:** body weight support device, spaceflight, locomotion, hypogravity, neurorehabiliation

## Terrestrial offloading

Whilst 30% Body Weight Support (BWS) unloading, via vertical suspension or Lower Body Positive Pressure (LBPP) systems, is used extensively on Earth for gait (walking) rehabilitation, it is equivalent to the 0.7 g return force provided to astronauts on the International Space Station (ISS) to allow them to walk or run on the T2 treadmill. There is general agreement that understanding movement generation and accuracy during terrestrial offloading may provide valuable insight into operations on the Moon and Mars (De Martino et al.). For example, individuals tend to trip and fall on Earth when traversing across uneven, unstable, or slippery ground—particularly when carrying an object. On the Lunar surface, Apollo crews fell when the ground was uneven and/or when interacting with or carrying a payload (1). Identifying the individual factors determining or metrics predicting such fall risk remains elusive on Earth, and this challenge is even greater on the Lunar surface, where a fall could be unrecoverable.

## Lunar locomotion

Although “bunny hopping” is often associated with Lunar locomotion, mission reports showed that astronauts adopted several locomotion styles, including skipping and loping (1). However, Lunar locomotion was not only influenced by the Moon's gravity and its surface characteristics but also by the restrictive Extra-Vehicular Activity suits, which limited mobility at the lower limb joints, and the Portable Life Support System backpack that displaced the crew's center of mass (CoM) posteriorly and vertically forcing them to lean forward (2), presumably in an attempt to retain the CoM within their base of support.

Unfortunately, a comprehensive assessment of gait biomechanics was impossible on the Lunar surface. However, several ground-based analogs have been employed, including ESA's Movement in Low Gravity (MoLo) programme that has evaluated simulated hypogravity locomotion during LBPP, then the Verticalised Treadmill Facility where participants are suspended horizontally with a multi-point harness and graded return forces generated toward the treadmill belt via pressure cylinders (3). Most recently, the MoLo programme employed the LOOP facility at the University of Milano (4) where a vertical suspension system using calibrated bungee cords provides near-constant unloading that appears to be more natural and, hence, more fluid movement—a feature of Apollo locomotion.

Data from the LOOP facility was employed to inform a novel computational framework for internal musculoskeletal loading and muscle adaptation estimation in hypogravity (Cowburn, Serrancolí, Pavei, et al.), which should consider the modulation of optimal fiber length (Cowburn, Serrancolí, Colyer, et al.) generated by long-term head-down bed rest—the key ground-based analog of microgravity. LBPP was used to unload locomotion, demonstrating a decrease in lower limb muscle activity, particularly the quadriceps and triceps surae (Fazzari et al.). In contrast, hamstring muscle activity increased during both the braking and push-off phases, likely counteracting the unloading-induced reduction of stride frequency (Fazzari et al.).

Interestingly, the tendency for neuromuscular adaptation during unloaded running appears to be related to trait anxiety (Fazzari et al.), suggesting psychological state may modulate the response to unloaded gait rehabilitation and could even play a role in the adoption of locomotion styles on the Moon. Perception may also modulate locomotion reported during parabolic flight-simulated hypogravity where, despite actual head contact being unlikely—the perceived proximity of the cabin roof may suppress vertical CoM displacement—a key characteristic of lunar locomotion. Furthermore, the relatively short period of hypogravity (≈30 s) is insufficient to generate a “stable gait”—which is required for many gait tests and is achieved during daily-life walking bouts.

## Lunar habitats

Crew movement within future Lunar habitats will present different challenges compared to floating around a space vehicle, including hypogravity locomotion within potentially confined (3D) spaces (potentially including insufficiently high ceiling structures—which requires definition) and complex stowage and handling tasks that require reaching. Indeed, reaching is provocative in 1 g, but it may be more challenging to perform safely in hypogravity. Lunar habitats are also likely to involve elements such as stairs or ladders to access storage/sleeping areas, move between habitat levels, and potentially move between the habitant and to the surface either due to the habitant either being raised from or partly buried in regolith. In fact, on Earth, stair climbing ability is considered a key risk factor for falls (5) and was a major concern of Apollo mission control—not least when Neil Armstrong took a “small step” down the Eagle Lander step ladder. Despite this, Apollo astronauts frequently jumped off the ladders due to the difficulty of descending while wearing bulky EVA suits, anecdotally reporting impatience rather than difficulty *per se*. This highlights the need for a clear definition of safe Lunar ergonomics and optimal habitat design.

An initiative that could help to address these challenges is the FlexHab element of the LUNA project, a large-scale Lunar analog facility currently being developed adjacent to the European Astronaut Center in Cologne. LUNA will simulate surface operations, including extra-vehicular activities, but could, with strategic use of co-located facilities, also form part of an operational ground-based simulation (Green and Drilea, under review).^1^ Whilst the dynamic offloading systems in LUNA aim to be compatible with the duration of proposed surface EVA operations—they should ideally also facilitate the investigation of intra-habitant ergonomics, such as stair climbing and ladder descent to inform operational concepts, inclusive design, and implementation of safeguards to support crew functionality and safety in Lunar habitats. However, such work could also be informative on Earth for individuals with functional sensory dysfunction that contributes to clumsiness and falls risk.

## Conclusion

Thus, investigating the risk factors for falls and locomotion in simulated hypogravity is critical not only to inform and de-risk life and work in lunar habitats—by modulating the effect of gravity and the relative effectiveness of righting responses—but also to provide novel data to inform terrestrial rehabilitation, in particular where dynamic postural control, including control of the CoM, is a key factor.

## Footnotes

^1^Green AD, Drilea I. Lunar life: an integrated multi-facility ground-based analogue of lunar surface missions. *Nat. Microgravity*. (under review).
